# A Close and Supportive Interparental Bond During Pregnancy Predicts Greater Decline in Sexual Activity From Pregnancy to Postpartum: Applying an Evolutionary Perspective

**DOI:** 10.3389/fpsyg.2019.02974

**Published:** 2020-01-10

**Authors:** Tierney K. Lorenz, Erin L. Ramsdell, Rebecca L. Brock

**Affiliations:** ^1^Department of Psychology and Center for Brain, Biology and Behavior, University of Nebraska-Lincoln, Lincoln, NE, United States; ^2^Department of Psychology, University of Nebraska-Lincoln, Lincoln, NE, United States

**Keywords:** pregnancy, postpartum, intimate relationships, sexual activity, parental investment, reproductive strategies

## Abstract

A common topic for advice given to parents after childbirth – both from relationship experts and popular media – is how to “bounce back” to one’s pre-pregnancy sexuality, with warnings that postpartum declines in sexual frequency will take a serious toll on one’s relationship. However, these admonishments may not accurately reflect the ways in which the unique reproductive context of pregnancy and the postpartum transition alter associations between sexual frequency and relationship quality. Evolutionary perspectives on reproductive strategies would suggest that in the postpartum context, decreased sexual activity would help target parental investment in the current offspring (rather than creating new offspring); however, if the parental relationship is lacking in intimacy and support, continued sexual activity may help seal the cracks in the bond. We tested this theory in a longitudinal dyadic study of changes in relationship quality and sexual frequency from pregnancy to 6 months postpartum among 159 heterosexual couples. We found that across three different measures of relationship quality taken from interviews and behavioral observation of couple interactions, *higher* relationship quality (i.e., greater support, intimacy, and responsiveness) predicted greater decline in sexual frequency whereas sexual frequency remained relatively stable in lower quality relationships. These findings suggest that, during the postpartum transition, decreased sexual frequency may not be a reliable signal of poor relationship quality.

## Introduction

Many parents-to-be wonder how the birth of their child will impact their relationship, including their sexual relationship. Although most parents expect some changes in their sexual lives, the nature of these changes, and what they may mean for the relationship more broadly, can be worrying. Does a strong bond during pregnancy predict more sex once the baby arrives – and does lack of sex predict future relationship dissatisfaction? Or is there something unique about the transition to parenthood that changes the typical associations between relationship quality and sexual frequency? Evolutionary theories predict that sexuality and partnership can take on different functions during this important reproductive transition, leading to unexpected outcomes: less sex after childbirth might *not* actually reflect relationship problems and, instead, could be a sign of successful adaptation to parenthood. Understanding how the birth of a child alters the patterns of associations between relationship quality and sexual activity will help parents better navigate a tricky transition and will inform evolutionary models of close relationships. In the present study, we examined how dyadic measures of relationship quality and sexual function during pregnancy predicted frequency of sexual activity 6 months postpartum.

Many parents experience a decline in frequency of sexual activity after the birth of a child. Indeed, most heterosexual couples show a gradual but significant decline in the frequency of sexual activity that starts during pregnancy and extends into the first year postpartum (Jawed-Wessel and Sevick, 2017). Additionally, approximately two-thirds of new mothers report changes in their sexual function, including desire, arousal and orgasm function (McBride and Kwee, 2017); it should be noted, however, that sexual function returns to pre-pregnancy levels for most women by 6 months postpartum (Connolly et al., 2005). Sexual desire, in particular, appears to decline for mothers (Rosen et al., 2018), with greater declines among breastfeeding vs. bottle-feeding mothers (LaMarre et al., 2003). Research on changes in sexual desire and/or erectile function among fathers across the perinatal period is more limited, with most work documenting his reactions to his partner’s changes in sexual desire (e.g., Schlagintweit et al., 2016). Similarly, the vast majority of studies examining changes in the sexual relationship among couples transitioning into parenthood have investigated mixed-sex (male/female) couples; the few studies of same-sex couples have largely found patterns that parallel that of mixed-sex couples, with significant declines in sexual activity (Goldberg et al., 2010; Huebner et al., 2012) and sexual function (van Anders et al., 2013) in the initial postpartum/post-adoption period.

Interestingly, although both parents typically report moderate distress regarding decreased sexual frequency and function, mothers are slightly more likely to report concern related to the decline in frequency of intercourse postpartum relative to fathers (Schlagintweit et al., 2016). This may reflect mother’s expectations that her own desire will follow from that of her partner (Hipp et al., 2012; Rosen et al., 2018), and subsequent concerns when his desire dips (Vannier et al., 2018). Moreover, when parents perceive changes in sexual frequency and function as stable – that is, that declines in either frequency or function are not simply due to the temporary state of pregnancy and postpartum – they are more likely to report relationship dissatisfaction (Vannier et al., 2018).

Parental distress regarding declining sexual frequency is grounded in very real concerns about the potential impact on the intimate relationship – which in turn, can influence the wellbeing of their child. In non-pregnancy contexts, a decline in sexual frequency is indeed associated with lower relationship quality and satisfaction (Litzinger and Gordon, 2005; Smith et al., 2011; McNulty et al., 2016) and can lead to relationship conflict and instability (Metz and Epstein, 2002; Hill et al., 2017). Recognizing that relationship conflict can interfere with creating a positive environment that supports healthy child development (Amato, 2001; Davies et al., 2006; Brock and Kochanska, 2016) authors across disciplines have encouraged clinicians specializing in postpartum care to counsel their patients regarding changes in sexual activity and sexual wellbeing (see e.g., Glazener, 1997; Leeman and Rogers, 2012; O’Malley et al., 2015). Similarly, popular media outlets abound with articles on how to “bounce back” to one’s pre-pregnancy body and sexuality, with the strong implication that the faster one returns to sexual activity, the healthier the parental relationship will be (Roth et al., 2012; Williams et al., 2017).

However, the assumption that sexual decline will always lead to problems in intimacy or decreased support from one’s partner does not match a growing literature investigating changes in sexual frequency across important life transitions. For example, work on sexuality in the context of aging suggests that although sexual frequency declines in older couples, the effect of these declines on relationship satisfaction depends on the couple’s efforts to communicate and connect with their partners (Forbes et al., 2017). In fact, declining sexual frequency may reflect the aging couple’s adjustment to each other’s preferences over time, a process the authors term “sexual wisdom.” Similarly, following significant illness or injury, many couples report an adjustment period in which their sexual frequency may decline to accommodate physical changes in one or both partners, but sexual quality improves as they expand their sexual repertoire or increase their mindfulness and emotional intimacy during sex (Gilbert et al., 2011; Fritz et al., 2015). It appears that, for couples undergoing a transition with concomitant changes in physical health status, decline in sexual frequency may or may not reflect poor relationship quality. To the extent that the relationship is of good quality– that is, providing both partners with emotional intimacy, social support, and effective communication – changes in sexual frequency may reflect healthy adjustment to the new realities of the couple’s situation.

Thus, what is missing in the handwringing over getting new parents back to their pre-pregnancy sexual patterns is consideration of whether or not sexual activity even *should* resume exactly as before – or if it is normal and adaptive for sexual activity to take on new functions in the postpartum context. Considered from an evolutionary perspective, the reproductive context of pregnancy and the postpartum transition present unique challenges which require significant adaptations in both parents (Chisholm et al., 1993; Kaplan, 1996) including, potentially, changes in sexual behavior.

As noted above, many studies show that declines in sexual frequency across the perinatal period can occur even among physically and mentally healthy parents. Decreasing sexual activity may represent an adaptive mechanism to decrease the chances of getting pregnant while caring for an infant – that is, avoiding investment in new offspring while one’s current offspring are still dependent (Rupp et al., 2013). This appears to be reflected in the significant neural and cognitive adaptations during pregnancy and postpartum leading to lower responsiveness to sexual stimulation but increased responsiveness to infant-related stimuli (Kim et al., 2010; Anderson and Rutherford, 2012; Rupp et al., 2013, 2014; Gregory et al., 2015). In fact, *lack* of decreased neural responsiveness to sexual stimuli can be a marker of postpartum depression (Lorenz et al., 2019).

On the other hand, even in the postpartum context, sexual activity still serves a role in maintaining the interparental bond – a bond that is especially vital to maintaining the health and well-being of individual family members and developing healthy parent-child dynamics (Erel and Burman, 1995; Ramsdell and Brock, under review). Again, taking an evolutionary perspective, mothers are particularly vulnerable during the months following childbirth and require significant support from their partners to improve their offspring’s survival (Geary, 2015). While it is in the father’s best interest to see his offspring survive, there may be more latitude in the degree of support he chooses to provide (Hagen, 1999; Geary, 2000; Hewlett, 2000). His investment in the newborn may be particularly tenuous if he doubts his partner’s sexual interest in him, which may raise concerns about paternity (Marlowe, 2000). In this context, sexual activity during the perinatal period may reflect a mother’s reassurance of sexual interest and attempts to maintain a strong and stable partnership in support of her offspring.

Taken together, these perspectives suggest that sexual frequency and relationship quality should have very different patterns of association during the postpartum transition than at other timepoints: the closer and stronger the bond between parents, the *lower* their sexual frequency as they are shifting focus to their new child. The less close the parental relationship, however, the more the couple will need to use sex as a means of maintaining paternal support and thus the higher their sexual frequency. We tested this hypothesis using a longitudinal study of heterosexual couples during pregnancy and 6 months postpartum, assessing multiple dimensions of interparental relationship quality and frequency of sexual activity. We predicted that markers of higher relationship quality (perceptions of emotional closeness and partner support, observed responsiveness and synchronicity) during pregnancy would predict a *greater* rate of decline in sexual frequency from pregnancy to the postpartum period. Given the literature documenting significant changes in sexual function among mothers during the postpartum transition, we also explored whether our predicted pattern of results would persist when accounting for mother’s sexual functioning. Specifically, we were interested in whether decline in sexual frequency was found even among mothers who reported high relationship quality *and* no significant sexual problems: if so, this would further support the hypothesis that decline in sexual activity during the postpartum transition is not (necessarily) a marker of poor sexual and relationship functioning.

Of note, we examined multiple indices of relationship *quality* rather than relationship *satisfaction* given that observed patterns and feelings of emotional closeness, partner support, and responsiveness have closer theoretic ties to the offspring’s survival than global feelings of contentment in one’s relationship (Geary, 2000). Similarly, we chose to focus on changes in sexual frequency as an outcome rather than changes in relationship satisfaction for two reasons. Firstly, as noted above, the decline in sexual frequency has been widely observed but not well theorized, leaving a gap in our understanding of these changes. That gap in understanding has been filled with recommendations to increase sexual frequency postpartum – potentially to the detriment of tired and stressed new parents. Secondly, from an evolutionary perspective, the neural mechanisms that coordinate sexual behavior are older and likely to be conserved in mechanisms coordinating relationship satisfaction. In other words, the factors that drive changes in sexual behavior likely form the foundation on which changes in other, more culturally determined factors such as relationship satisfaction would occur.

## Materials and Methods

### Participants and Procedures

Flyers and brochures were broadly distributed in the (Lincoln and Omaha) metropolitan area to businesses and clinics frequented by pregnant women and, if an establishment permitted, we approached potential participants and provided a brief overview of the study. Eligibility criteria included: (a) 19 years of age or older (legal age of adulthood where the research was conducted), (b) English speaking, (c) mother was pregnant at the time of the initial appointment, (d) both partners were biological parents of the child, (e) singleton pregnancy (i.e., not twins or multiples), and (f) in a committed intimate relationship and cohabiting.

One hundred sixty-two cohabitating couples who were expecting a child were enrolled in the study. Three couples were excluded from the final sample, due to either invalid data or ineligibility, for a final sample of 159 couples (159 women and 159 men). Couples had dated an average of 81.90 months (*SD* = 49.59), cohabited an average of 61.00 months (*SD* = 41.80) and the majority of couples were married (84.9%). Over half (57.8%) reported that they had no children (i.e., first-time parents). Most women were in the second (38.4%) or third (58.5%) trimester of pregnancy. Participants were primarily White (89.3% of women; 87.4% of men); 9.4% of women and 6.4% of men identified as Hispanic or Latino. On average, women were 28.67 years of age (*SD* = 4.27) and men were 30.56 years of age (*SD* = 4.52). The sample reported a median *joint* income of $60,000 to $69,999, and most participants were employed at least 16 h per week (74.2% of women; 91.8% of men). Further, the modal education was a bachelor’s degree (46.5% of women; 34.6% of men).

During pregnancy, both partners attended a 3-h laboratory appointment during which they completed behavioral observation tasks and semi-structured clinical interviews about the quality of their intimate relationships. Partners were escorted to separate rooms to complete the clinical interviews and self-report questionnaires and did not interact with one another until the procedures were complete. Participants were compensated with $50 (for a total of $100 per couple) for attending the appointment.

At a time scheduled for 6 months postpartum (actual time: *M* = 6.32 months, *SD* = 0.36), each parent completed a phone interview with a member of the research team (separately and in a private location) during which they responded to questions about the quality of their sexual relationship since the baby was born. Each participant received $25 for the 6-month postpartum assessment (for a total of up to $50 per couple). All procedures were approved by the university’s Institutional Review Board, and all participants provided informed consent.

### Measures of Interparental Relationship Quality During Pregnancy

#### Semi-Structured, Objectively Coded Interviews

The *Relationship Quality Interview* (RQI; Lawrence et al., 2009, 2011) is a 60–90 min interview designed for interviewers to conduct functional analyses of intimate relationships across multiple domains; to assess core dimensions of interparental relationship quality for the present study, we examined emotional intimacy and partner support (see below for detailed descriptions). The RQI has demonstrated excellent reliability, convergent validity, and divergent validity (Lawrence et al., 2009, 2011). Open-ended questions, followed by closed-ended questions, were asked to obtain novel contextual information about functioning in the relationship during the past six months. Concrete behavioral indicators were used to facilitate objective ratings; for example, “how often do you confide in your partner, or disclose personal important things to him/her?” and “how does your partner respond when you disclose?”. As such, the RQI is not a measure of individual perceptions of relationship satisfaction but, rather, assesses functioning in the relationship. Interviewers independently rated emotional intimacy and partner support in the participant’s relationship on a scale of 1 (poor functioning) to 9 (high functioning). Partners were interviewed separately and simultaneously to prevent response contamination.

Interviewers completed training in reliable coding of the RQI and participated in consensus and recalibration meetings throughout the course of the assessment period to ensure reliable coding. A different research assistant coded each partner (from the same couple), and coders were instructed not to discuss interviews from the same couple, to ensure maximum objectivity. Approximately 20% of the maternal and paternal interviews were randomly assigned and double-coded to assess interrater reliability, which was excellent (average ICC = 0.91).

#### Emotional Intimacy

From the RQI, the domain of emotional intimacy is assessed by considering the following: the degree to which partners of a dyad engage in emotionally intimate interactions, experience a sense of mutual closeness, warmth, and interdependence, feel comfortable being emotionally vulnerable with one another, engage in high-quality emotional disclosure, and demonstrate love and affection toward one another. Scores from maternal and paternal interviews were significantly correlated (*r* = 0.26, *p* = 0.001), and, as such, were aggregated to create a dyadic score.

#### Received Partner Support

Also from the RQI, the domain of partner support is assessed by considering the following: the quality of support in response to stress (e.g., listening, providing advice, taking care of things directly or indirectly, spending time together, boosting confidence), match between desired and received levels of support, and whether support is offered in a positive/negative manner. In this sample of pregnant couples, the inter-partner correlation between maternal and paternal scores of received support was non-significant (*r* = 0.11, *p* = 0.161). Closer examination of mean differences in scores from interviews with each partner revealed that, on average, fathers received higher quality support from their partners (relative to mothers), *t*(158) = 2.05, *p* = 0.042. Because of gender differences in this sample of pregnant couples, and the small correlations between objective ratings from interviews with each partner, we ultimately examined separate partner scores of support, but tested them simultaneously in the same model.

#### Observed Relationship Quality

To obtain a measure of interparental mutually responsive orientation (MRO), the interparental dyad was observed for approximately 30 min, in standardized, naturalistic, interactive contexts during the laboratory session conducted during pregnancy. The contexts included 3 10-min discussions about planning a vacation together, something the mother would like to change about herself, and something the father would like to change about himself.

A team of four coders viewed the video interactions of interparental dyads during the aforementioned contexts and coded the couple’s MRO: that is, the degree to which both members of the dyad have established a system of reciprocity, coordination, and synchronicity. The coders considered four dyadic dimensions: coordinated routines (couple works well together), harmonious communication (smooth “back and forth” nature to conversation), mutual cooperation (responsivity to one another), and emotional ambiance (engaged; showing positive emotion). Importantly, coders also considered observations of negative interactions (not simply absence of positive interactions) in their final ratings; for example, in the harmonious communications dimension, coders not only included lack of communication but also evidence of hostility. Interactions were coded on a 5-point scale for each of the three observed contexts, from 1 = *poor relationship* to 5 = *excellent relationship*, and then scores were aggregated across the three contexts for one overall judgment of interparental MRO. MRO scores have demonstrated adequate interrater reliability (ICCs: 0.77 for maternal support task, 0.82 for paternal support task, and 0.71 for vacation task) and excellent convergent and divergent validity (Brock et al., under review).

### Measures of the Sexual Relationship During Pregnancy and Postpartum

#### Frequency of Sex During Pregnancy and Postpartum

In addition to measuring emotional intimacy and partner support, the RQI also assesses multiple features of the couple’s sexual relationship and produces a score of frequency of sex during the past 6 months. Specifically, participants are asked “*About how frequently do the two of you have sex*?” The interviewer assigned a score using the following rating scale: 1 = rare or not at all, 2 = less than once per month, 3 = once or twice per month, 4 = once per week, 5 = multiple times per week. The RQI was also administered at 6 months postpartum and, once again, partners reported on the frequency of sex in their relationship over the past 6 months (i.e., since the baby was born). Scores between partners of the same dyad were significantly correlated at both the pregnancy timepoint (*r* = 0.53, *p* < 0.001) and at 6 months postpartum (*r* = 0.60, *p* < 0.001), showing relative agreement about the frequency at which they are having sex as a couple. As such, maternal and paternal scores were averaged to create dyadic scores of sexual frequency during pregnancy and at 6 months postpartum.

#### Maternal Sexual Problems During Pregnancy

Sexual problems among mothers during pregnancy were also coded from the RQI to examine as a covariate. To gather information about sexual problems, mothers and fathers were asked if they or their partner have experienced any sexual difficulties in the past 6 months. If so, participants were invited to elaborate on the nature of the difficulty. Mothers were considered to be experiencing sexual problems if they or their partners noted that she had experienced one or more of the following: vaginal dryness, delayed orgasm, inability to achieve orgasm, or decreased sexual desire. Sexual problems were coded dichotomously (0 = no dysfunction reported, 1 = at least one symptom noted). Approximately 20.8% of mothers had experienced some form of sexual dysfunction during pregnancy. In the present study, we considered only mother’s sexual problems, as the literature on sexual function during pregnancy and postpartum most clearly documents changes among mothers (see e.g., Jawed-Wessel and Sevick, 2017). It should also be noted that participants were not specifically asked about distress regarding these symptoms (although many did express distress), and thus we could not establish criteria for diagnosis of sexual dysfunction *per se* (Shifren et al., 2008); these data should instead be interpreted as emergence of sexual problems that may (or may not) merit clinical attention.

### Data Analytic Plan

**FIGURE 1:**
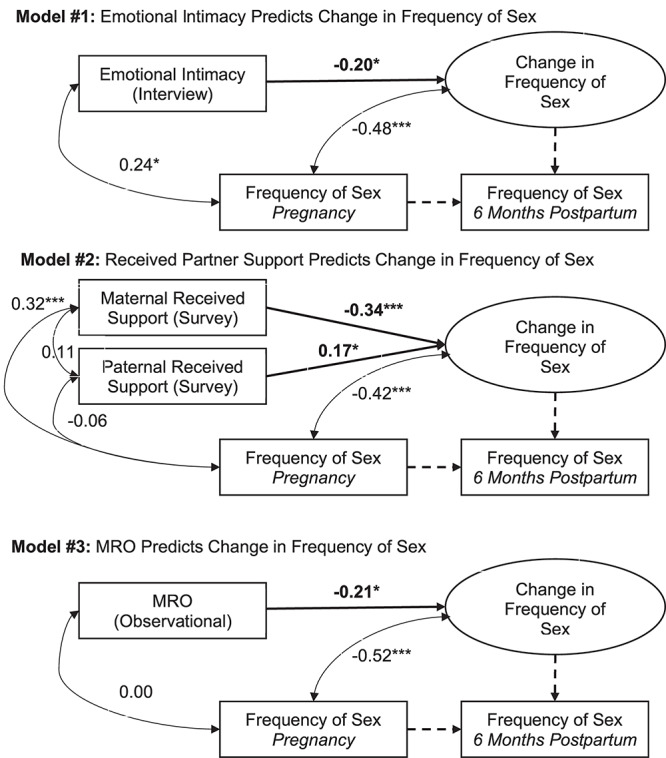
Statistical models and results. Tests of study hypotheses are bolded. Parameters fixed to 1.00, as required to model the latent change score variable, are dashed. Standardized coefficients for freely estimated paths are reported. ^∗^*p* < 0.05. ^∗∗∗^*p* < 0.001. MRO: Mutually Responsive Orientation.

Data were analyzed using Mplus software (Muthén and Muthén, 2010). Missing data were minimal (covariance coverage ranged from 0.97 to 1.00), and were addressed with Full Information Maximum Likelihood (FIML) estimation. To account for violations of univariate and multivariate normality, MLR was used to obtain robust standard errors. The models were just identified; therefore, global model fit was not assessed. To examine change in the frequency of sex from pregnancy to the postpartum period, we applied a latent change score framework (McArdle and Nesselroade, 1994; Castro-Schilo and Grimm, 2018). Within this model, a latent variable is estimated which represents a within-person change score (i.e., the degree of change from pregnancy to 6 months postpartum) that can vary across participants. Model specification is depicted in Figure 1. Subsequently, the latent change score was regressed on (1) emotional intimacy (measured via semi-structured interview), (2) partner support (measured via semi-structured interview), and (3) mutually responsive orientation (measured via behavioral observation). In the case of the support model, given that partner support scores were individual (rather than dyadic), the latent change score was regressed on both maternal and paternal reports of received support.

Prior to conducting analyses, we assessed for any differences between first vs. repeated parents on all dimensions of relationship quality. Correlations between first time parenthood status (1 = no children in the home, 0 = at least one child in the home during pregnancy) and the relationship quality predictors (emotional intimacy, received partner support, and mutually responsive orientation) were small and non-significant (*r*’s ranged from −0.045 to 0.12, *p*-values > 0.05). Additionally, first time parenthood status was not significantly associated with change in sexual frequency (*B* = 0.043, *p*-value > 0.05). As such, we did not include parenthood status as a control variable in the model.

## Results

Descriptive statistics and correlations among the study variables are reported in Table 1. Results of the present study are depicted in Figure 1 and reported in Table 2. Consistent with past research (Jawed-Wessel and Sevick, 2017), a latent change score model (omitting predictors) demonstrated that, on average, frequency of sex decreased from pregnancy to 6 months postpartum, *M* of the latent change score variable was −0.21, *p* = 0.02. Additionally, there was significant variance in the latent change score variable (1.04, *p* = 0.00) suggesting that not every couple experienced the same magnitude (or direction) of change.

**TABLE 1:** Correlations and descriptive statistics.

	**1**	**2**	**3**	**4**	**5**	**6**
(1) Emotional Intimacy	–					
(2) Maternal Received Support	0.60^∗∗^	–				
(3) Paternal Received Support	0.43^∗∗^	0.11	–			
(4) Mutually Responsive Orientation (MRO)	0.30^∗∗^	0.09	0.25^∗∗^	–		
(5) Frequency of Sex During Pregnancy	0.24^∗∗^	0.32^∗∗^	–0.06	0.01	–	
(6) Frequency of Sex 6 Months Postpartum	0.07	0.10	0.07	–0.15	0.53^∗∗^	–
Mean	6.92	6.56	6.82	3.47	3.73	3.49
*SD*	0.85	1.34	1.08	0.85	1.06	1.03
*N*	159	159	159	152	159	135

*^∗∗^*p* < 0.01.*

**TABLE 2:** Model results.

**Model**	**Unstandardized Coefficient**	***SE***	***p-value***
**Model #1: Emotional Intimacy**			
Emotional Intimacy Predicting Change in Frequency of Sex	**−0.240**	**0.110**	**0.020**
**Model #2: Received Partner Support**			
Maternal Received Support Predicting Change in Frequency of Sex	**−0.257**	**0.066**	**0.000**
Paternal Received Support Predicting Change in Frequency of Sex	**0.159**	**0.065**	**0.014**
**Model #3: Mutually Responsive Orientation (MRO)**			
MRO Predicting Change in Frequency of Sex	**−0.250**	**0.110**	**0.030**

*The model results reported above exclude sexual function, given the interactions between sexual function and relationship quality (i.e., emotional intimacy, support, and MRO) were non-significant. Significant effects (*p* < 0.05) are bolded.*

Results provide support for our hypothesis that an intimate, supportive, and responsive relationship during pregnancy would predict decline in frequency of sex from pregnancy to 6 months postpartum. Couples experienced more decline in frequency of sex to the extent that (a) couples had a higher degree of emotional intimacy, as measured via semi-structured interviews with both partners (see Figure 1, Model 1), (b) mothers received higher quality support from their partners during pregnancy; whereas, interestingly, the extent to which fathers reported receiving higher quality support from their partners during pregnancy was actually associated with *less* decline in frequency of sex (see Figure 1, Model 2), and (c) couples displayed a more mutually responsive orientation during observed interactions (see Figure 1, Model 3). The patterns of results were unchanged when including mother’s sexual functioning, and the interaction between mother’s sexual functioning and relationship quality, in the model (moderation effects ranged from −0.03 to 0.24 across the models, *p*-value’s > 0.05).

## Discussion

### Discussion of Main Findings

The transition to parenthood can be both exciting and confusing, presenting both parents with the challenges of adopting new roles and new ways of relating to each other while supporting their child. Sexuality and intimacy can take on new meaning during this transition, leaving many parents wondering what is normal and what might signal problems ahead. We followed couples during pregnancy and 6 months postpartum, measuring changes in their frequency of sexual activity and relationship quality using multimodal measures. Those couples who reported greater emotional closeness and responsiveness in their relationship during pregnancy had a *greater* decline in sexual frequency than those who reported less intimate and synchronous relationships. Notably, when considering the impact of partner support on changes in sexual frequency, there were gender differences. Specifically, there were greater declines in sexual frequency when mothers received higher quality support from their partners whereas decline in sexual frequency was predicted by fathers receiving *poorer* quality support. Interestingly, the aforementioned patterns remained significant even when considering the impact of sexual problems on sexual frequency.

While at first glance it may seem surprising that *higher* interparental relationship quality is associated with greater decline in sexual activity, this finding fits predictions from evolutionary models of close relationships during an important reproductive transition. The decline in sexual frequency observed in couples who had closer bonds may reflect a positive adaptation, delaying further investment in new offspring (Gray, 2013; Rupp et al., 2013), and reducing potential costs of postpartum sex such as infection or pain (Lehtonen et al., 2012). When the couple has a strong and trusting bond, their energy can be directed at navigating the challenges of parenthood: sex can take a back seat for a little while. But when new parents perceive a lack of intimacy and responsiveness, then they may turn to sex as a means of shoring up their troubled bond (Magginetti and Pillsworth, 2019). This is reflected in the relatively higher number of pregnant and postpartum women who report engaging in sex for relationship maintenance reasons than for sexual desire or pleasure reasons (Gerda et al., 2006; Bello et al., 2011; Hipp et al., 2012; Sagiv-Reiss et al., 2012).

In particular, when new mothers perceive a lack of support from their male partners, they may engage in sex more frequently to renew paternal investment in both the relationship and the offspring. Indeed, other studies have suggested that when there are threats to the relationship (such as perceptions of other women flirting with their husband), pregnant women’s sexual desire for their partner paradoxically increases (Magginetti and Pillsworth, 2019). Insofar as sexual activity represents a possible investment in a new child, while decline in sexual frequency signals the need to consolidate resources in the current offspring, we would expect different patterns in mothers versus fathers based on their relative investment in reproduction more generally. Indeed, we saw some evidence of gender differences in the association between perceived support and decline in sexual activity. Simply put, fathers have (relatively) more leeway in the degree of support they provide, and the investment they make during the postpartum period (Gray, 2013). Accordingly, when support received by mothers is of poor quality, mothers may have to negotiate greater support by engaging in more sexual activity; when support received by mothers is of high quality, she can afford to redirect her energies to her offspring. On the other hand, when mother’s support of the father is of low quality, he will likely not improve her support by seeking more sex: if anything, this would further tax the mother’s resources and make her less inclined to provide him support.

Most previous research on intimate relationships has taken the perspective that decline in sexual frequency is a negative outcome, and would be expected in relationships that are less intimate, supportive, and mutually responsive. However, our findings suggest quite the opposite when a couple is caring for a newborn. At a proximal level of analysis, this contrast is likely tied to the different factors that lead to lower sexual frequency in parents and non-parents: whereas non-parents are mostly likely to report declining sexual frequency stemming from relationship conflict and sexual dysfunction (Laumann et al., 1999; Sprecher et al., 2004), parents navigating the postpartum period are more likely to cite fatigue (Ahlborg et al., 2005; Hipp et al., 2012), lack of time, and concerns about causing harm to the mother’s body after childbirth (Schlagintweit et al., 2016; Beveridge et al., 2018). In other words, the negative effects of decline in sexual frequency may be buffered by perceptions that the decline is situational and temporary (Vannier et al., 2018), and feeling that avoiding sex is an expression of care and commitment to one’s partner. For example, in one study, mothers who reported their partner was understanding of their need to avoid or delay sex had significantly higher relationship and sexual satisfaction (Muise et al., 2017). Another proximate mechanism may be cognitive dissonance. That is, it is possible that couples with lower relationship quality may engage in more frequent sex even after the postpartum transition, in order to resolve the psychological stress of being committed to a less intimate and supportive relationship, when they are forced into this commitment by virtue of having to rely on each other to care for the newborn.

### Discussion of Secondary Analyses

We also examined how relationship quality predicted change in sexual frequency in women with and without sexual problems, and found no significant difference between these groups. In non-parenting contexts, relationship quality and sexual dysfunction often interact to predict declines in sexual frequency: when relationship quality is good, sexual dysfunction may or may not result in lower rates of sexual activity (Schoenfeld et al., 2017) but when relationship quality is poor, sexual function is strongly associated with sexual frequency (Litzinger and Gordon, 2005; Hayes et al., 2008). However, in the present study, including mother’s sexual problems in the model did not alter the associations between relationship quality and changes in sexual frequency during the postpartum transition. As with all null effects, it is important not to over-interpret the lack of an observed significant effect; it is possible that this analysis was underpowered to detect small, but potentially meaningful, effects. However, it is also possible that in the postpartum context, changes in sexual function may be more independent of relationship factors (Hansson and Ahlborg, 2012). Although a large number of studies have shown declines in sexual function in pregnant and postpartum mothers (see Jawed-Wessel and Sevick, 2017; McBride and Kwee, 2017 for reviews), these declines may be less related to relationship conflict and more closely aligned with other factors such as changes in the mother’s body [e.g., vascular changes in the vagina and clitoris following pregnancy (Battaglia et al., 2018) or perineal injury (Signorello et al., 2001)], hormonal factors [e.g., increased prolactin during breastfeeding (LaMarre et al., 2003)], fatigue (Schlagintweit et al., 2016), and pre-pregnancy sexual dysfunction (Yıldız, 2015). Taken together, these findings suggest that lower sexual functioning among parents of newborns may be less diagnostic of relationship problems than a consequence of the physical and lifestyle changes associated with parenthood.

### Clinical and Theoretic Implications

Our findings have several important clinical and theoretic implications. Firstly, when counseling parents on the resumption of sexual activity following childbirth, providers should consider setting expectations that a decline in sexual frequency is common, even more so among healthy couples. This is not to say that couples should be counseled to avoid sexual activity *per se*, but rather not to interpret decline in sexual frequency as a harbinger of larger relationship difficulties. New mothers in particular appear to be concerned about how changes in the sexual relationship will impact their partners (Schlagintweit et al., 2016), and may benefit from her partner’s encouragement to communicate when she needs to take a break from sex (Muise et al., 2017).

More broadly, these findings suggest that the reproductive context may change the nature of the association between interparental relationship dynamics and sexual activity. An important follow-up to the present study will be to investigate the tipping point at which the effect reverts, and low sexual frequency again signals relationship problems. If our hypothesis is correct, and the decline in sexual frequency follows the need to reallocate resources to a vulnerable offspring, we should expect sexual frequency to rise as the child grows and requires less direct parental support. If sexual activity does not resume even after the reproductive context shifts back to fertility, it may at that time indicate relationship strain. Additionally, as lactation is one of the strongest signals to the mother of the infants continued need for support (Vitzthum, 1994), we may expect that the tipping point will occur later for breastfeeding vs. bottle-feeding mothers.

Moreover, if the postpartum decline in sexual frequency reflects an adaptive response, temporarily shifting reproductive investment to the current offspring, we must question how we consider sexual desire within the construct of postpartum depression. Specifically, our findings call for the need to consider the reproductive context in which low desire occurs in order to determine if it contributes to evidence of psychopathology. Certainly, low sexual desire is common among postpartum depressed mothers (Khajehei et al., 2015) – but as it is common among healthy postpartum women as well (Chivers et al., 2011), the symptom may lack discriminant validity in this context.

### Limitations

While this study had significant strengths, including a longitudinal design and multimodal, dyadic measurement of both relationship quality and sexual frequency, there are some limitations worth noting. Participation in this study was limited to mixed-sex (heterosexual) couples, and thus the effects in same-sex couples, or non-dyadic parental structures (e.g., poly families) remain unclear. Future study of sexuality within non-traditional family structures will improve generalizability as well as contribute to the understanding of the evolution of alloparenting (Bogin et al., 2014). The present study sample was mostly White and middle class. Both minority stress and socioeconomic status are likely to increase the strength of the effects we observed: insofar as the postpartum decline in sexual frequency represents a rebalancing of resources from potential future offspring to the current offspring, we should expect stronger effects as couples are put under greater stress or financial hardship.

Our measure of sexual frequency was limited to partnered sexual activity; while this does not change our ability to test our main hypotheses, future studies may benefit from teasing apart changes in sexual activity (including solitary sexual activity) from changes specific to the sexual relationship. Similarly, we focused on mother’s sexual problems, as there was greater prior research on changes in women’s sexual function during pregnancy and postpartum; however, future work may benefit from consideration of changes in paternal sexual function as well. Also, our measure of sexual problems came from holistic participant self-report, which may or may not have included explicit discussion of sexual satisfaction or distress regarding their sexual problems. As noted above, this means that we cannot conclude these sexual problems would meet clinical criteria for a sexual dysfunction, as distress is a crucial element of the diagnosis. More precise measurement of sexual functioning and relationship satisfaction, which prompts participants to consider their distress, may reveal subgroups for whom sexual problems are a more significant moderator.

Finally, in interpreting these findings, it must be noted that we were primarily interested in the constructs most closely related to how the interparental relationship contributes to potential offspring survival, and as such we focused on indices of support, responsiveness, and intimacy rather than relationship satisfaction or happiness. There is a broad literature documenting declines in sexual and relationship satisfaction during the postpartum transition (see Mitnick et al., 2009 for review and meta-analysis), and their association with decline in sexual frequency (Schlagintweit et al., 2016; Rosen et al., 2018). While the link between relationship satisfaction and offspring survival is more indirect, it is certainly worth investigating if and how the present evolutionary framework may explain how longitudinal changes in parental attitudes toward the relationship map onto changes in sexual frequency.

## Conclusion

Couples often experience a decline in frequency of sexual activity from pregnancy to postpartum, and results of the present study suggest that this change is actually greater to the extent that couples have a close and supportive bond during pregnancy. This pattern is consistent with the principles of parental investment theory (Chisholm et al., 1993; Kaplan, 1996): lower sexual frequency may reflect better, not worse, adaptation to the unique reproductive context of parenthood by shifting resources toward the offspring. These findings suggest the need to reconsider how strongly we encourage new parents – particularly new mothers – to “bounce back” to their pre-pregnancy sexual habits as quickly as possible, or else suffer damage to their partnership. Rather, new parents should be reassured that less sex does not necessarily indicate personal or relationship deficits. More broadly, these findings point to the utility of evolutionary frameworks in understanding the role of sexuality in close relationships, and suggest the need for consideration of reproductive context when evaluating changes in a couple’s sexual relationship.

## Data Availability Statement

The datasets for this article are not publicly available because they contain sensitive and unavoidably identifiable information about participants (such as taped observations of interactions), and participants did not grant permission for these data to be shared publicly. Requests to access the datasets should be directed to RB at rebecca.brock@unl.edu.

## Ethics Statement

The studies involving human participants were reviewed and approved by the University of Nebraska–Lincoln Institutional Review Board. The patients/participants provided their written informed consent to participate in this study.

## Author Contributions

RB was responsible for the design and conduct of the original study from which this secondary analysis was drawn. TL contributed the specific hypotheses tested in this manuscript, including contextualization in the evolutionary psychology framework, and drafted the Introduction and Discussion sections. ER and RB designed and conducted the statistical analyses, and drafted the tables and figure, the Materials and Methods and Results sections. All authors contributed equally to revisions toward the final draft.

## Conflict of Interest

The authors declare that the research was conducted in the absence of any commercial or financial relationships that could be construed as a potential conflict of interest.
