# Corrigendum: A Close and Supportive Interparental Bond During Pregnancy Predicts Greater Decline in Sexual Activity From Pregnancy to Postpartum: Applying an Evolutionary Perspective

**DOI:** 10.3389/fpsyg.2020.01161

**Published:** 2020-06-03

**Authors:** Tierney K. Lorenz, Erin L. Ramsdell, Rebecca L. Brock

**Affiliations:** ^1^Department of Psychology and Center for Brain, Biology and Behavior, University of Nebraska-Lincoln, Lincoln, NE, United States; ^2^Department of Psychology, University of Nebraska-Lincoln, Lincoln, NE, United States

**Keywords:** pregnancy, postpartum, intimate relationships, sexual activity, parental investment, reproductive strategies

In the original article, there was an error. It was stated in the **Materials and Methods** section, that the modal education was a bachelor's degree in 34.6% of women. This was actually referring to men. A correction has been made to the Materials and Methods section, subsection **Participants and Procedures**, paragraph 2:

“One hundred sixty-two cohabitating couples who were expecting a child were enrolled in the study. Three couples were excluded from the final sample, due to either invalid data or ineligibility, for a final sample of 159 couples (159 women and 159 men). Couples had dated an average of 81.90 months (SD = 49.59), cohabited an average of 61.00 months (SD = 41.80) and the majority of couples were married (84.9%). Over half (57.8%) reported that they had no children (i.e., first-time parents). Most women were in the second (38.4%) or third (58.5%) trimester of pregnancy. Participants were primarily White (89.3% of women; 87.4% of men); 9.4% of women and 6.4% of men identified as Hispanic or Latino. On average, women were 28.67 years of age (SD = 4.27) and men were 30.56 years of age (SD = 4.52). The sample reported a median joint income of $60,000 to $69,999, and most participants were employed at least 16 h per week (74.2% of women; 91.8% of men). Further, the modal education was a bachelor's degree (46.5% of women; 34.6% of men).”

The authors apologize for this error and state that this does not change the scientific conclusions of the article in any way. The original article has been updated.

